# Myelodysplastic Syndrome with Transfusion Dependence Treated with Venetoclax

**DOI:** 10.1155/2020/9031067

**Published:** 2020-03-12

**Authors:** Waqas Jehangir, Alexander Karabachev, Taimoor Jahangir, Elvira Umyarova

**Affiliations:** ^1^University of Vermont Medical Center, Hematology and Medical Oncology, 89 Beaumont Ave., Burlington, VT 05405-0068, USA; ^2^University of Vermont College of Medicine, Larner College of Medicine. 89 Beaumont Ave., Burlington, VT 05405-0068, USA; ^3^University Hospitals of Leicester NHS Trust, Groby Rd., Leicester LE3 9QP, UK

## Abstract

Myelodysplastic syndromes are characterized by ineffective hematopoiesis in one or more lineages of the bone marrow. They are a group of heterogeneous clonal stem cell malignancies with a high risk to progress to acute myeloid leukemia. Currently, there are no curative FDA-approved medications for myelodysplastic syndromes. Hematopoietic cell transplantation is potentially the only curative option; however, treatment is often unavailable due to age and comorbidities. Hypomethylating agents, azacitidine and decitabine, and the immunomodulatory agent, lenalidomide, are the only FDA approved medications for the treatment of MDS, all of which are noncurative. Venetoclax, an inhibitor of the antiapoptotic protein BCL-2 used to treat chronic lymphocytic leukemia, is currently being evaluated in clinical trials as a monotherapy in high-risk myelodysplastic syndromes/acute myeloid leukemia. We present a patient with transfusion-dependent myelodysplastic syndromes refractory to the current standard of care treatment not a candidate for hematopoietic cell transplantation who responded well to monotherapy treatment with venetoclax and has since remained transfusion-independent.

## 1. Introduction

Myelodysplastic syndromes (MDS) are characterized by ineffective hematopoiesis in one or more lineages of the bone marrow. They are a group of heterogeneous clonal stem cell malignancies with a high risk to progress to acute myeloid leukemia (AML). Majority of adult MDS cases arise *de novo*; however, some are due to genotoxic damage from ionizing radiation or chemotherapy treatments [1]. In the United States, the incidence of MDS is 3-4 cases per 100,000 population per year and increases with age with 30 cases per 100,000 population per year among individuals of 70 years of age and older [2]. The prevalence of MDS is growing in part due to an ageing population and an increased awareness of these syndromes [3]. Prognosis of MDS is highly variable based on risk stratification ranging from several years in low-risk patients to several months in patients with high-risk disease [4].

Currently, there are no curative FDA-approved medications for MDS. Hematopoietic cell transplantation (HCT) is potentially the only curative option; however, treatment with HCT is often unavailable due to age and comorbidities or is not tolerated due to potential side effects. Hypomethylating agents (HMA), Azacitidine and Decitabine, and the immunomodulatory agent Lenalidomide are the only three FDA-approved drugs for the treatment of MDS, all of which are noncurative [5–7].

Venetoclax, a small molecule inhibitor of the antiapoptotic protein BCL-2, has demonstrated promising results as a monotherapy in high-risk MDS/AML in *in vitro* studies [8, 9] and is currently being evaluated in clinical trials as a single-agent and in combination with Azacitidine for relapsed/refractory MDS. In this case, we describe a patient with transfusion-dependent myelodysplastic syndrome refractory to the current standard of care treatments and not a candidate for hematopoietic cell transplantation who responded well to monotherapy treatment with Venetoclax and has since remained transfusion-independent.

## 2. Case Presentation

A 53-year-old male with past medical history of hypertension, hyperlipidemia, peptic ulcer disease, gout, coronary artery disease, and sleep apnea underwent a CABG procedure in December 2011. Following CABG, the patient's blood counts remained low, and in March of 2012, he was referred to Hematology at the VA for further evaluation. His CBC at that time demonstrated pancytopenia with a white count of 2100 × 10^6^/L, hemoglobin of 9.7 × 10^9^/L, and platelet count of 123,000 per microliter. He had 3% circulating blasts. The bone marrow demonstrated a hypercellular marrow with multilineage dysplasia. Cytogenetics were significant for translocation between chromosome 2 and 11 t(2; 11)(p21; q23). At that time, he was diagnosed with MDS (refractory anemia with excess blasts, intermediate 1) and was found to have a revised IPSS (International Prognostic Scoring System) score of 3. The initial bone marrow blast count was 4%, and the cytogenics was 46, XY, t(2; 11). A repeat bone marrow showed a blast count of 3%, and the cytgenics was 46, XY, t(2; 11).

The patient was started on Azacitidine in September of 2012. This was complicated by elevated liver function tests as well as prolonged neutropenia. The bilirubin peaked at approximately 5-6 mg/dL. In November 2012, he received a 20% dose reduction of Azacitidine which was again complicated by acute hepatitis and renal failure with a creatinine of approximately 6 mg/dL, a total bilirubin peaked at 10 mg/dL and AST and ALT of 401 and 414 units per liter. His CBC demonstrated pancytopenia, however, was stable and he had not required any further blood transfusions. The licensed indication for the use of Azacitidine in Europe is IPSS Int-2 and high; therefore, its use was off-label. He had a response to Azacitidine for almost 10 months. In May 2013, CBC showed a white blood cell count of 4,100 × 10^6^/L with a hemoglobin of 13.9 g/dL and a platelet count of 161,000 per microliter. His differential indicates 56% segs and 37% lymphocytes. There was no evidence of circulating blasts. His repeat bone marrow biopsy revealed normocellular with persistent myelodysplasia, Grade 1 myelofibrosis, and cytogenetics 46, XY t(2; 11) in 100%. The diagnostic bone marrow analysis showed Grade 2 myelofibrosis.

Given acute hepatitis, acute renal failure, and prolonged cytopenias, he was not felt to be good candidate for continuation of Azacitidine. Given his worsening cytopenias, low dose lenalidomide 5 mg PO every other day was initiated in Dec 2013 and then stopped for prolonged cytopenia. Its use was off-label, given the patient did not have del (5q). In Feb 2014, repeat bone marrow biopsy for declining counts was performed (WBC 2.7 × 10^9^/L, hemoglobin 12.3 g/dL and platelets 117,000 per microliter) which showed no change when compared to October 2012. He was evaluated at Dartmouth Hitchcock Medical Center for allogenic stem cell transplantation (alloHSCT) and had a 10/10 donor (sister) and, however, considered ineligible due to comorbidities of coronary artery disease and unstable angina. He remained on active surveillance until August 2018 when he became transfusion-dependent. He was on almost twice weekly transfusions through the VA. Bone marrow biopsy in August 2018 showed myelodysplastic syndrome: refractory cytopenia with multilineage dysplasia. On Novemeber 28, 2018, the patient was started on single-agent Venetoclax 100 mg daily. The use of Venetoclax was off-label and obtained through the compassionate use program through the VA.

**Figure 1:**
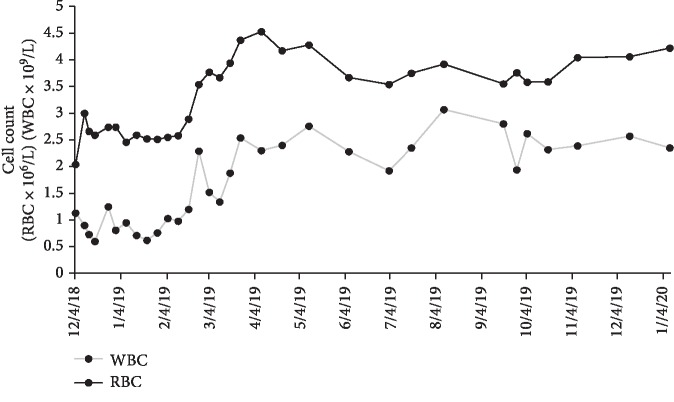
Red blood cell and white blood cell count recorded during Venetoclax monotherapy treatment from December 2018 to January 2020. The treatment initially began on November 28, 2018 at 100 mg and after one month was increased to 200 mg.

**Figure 2:**
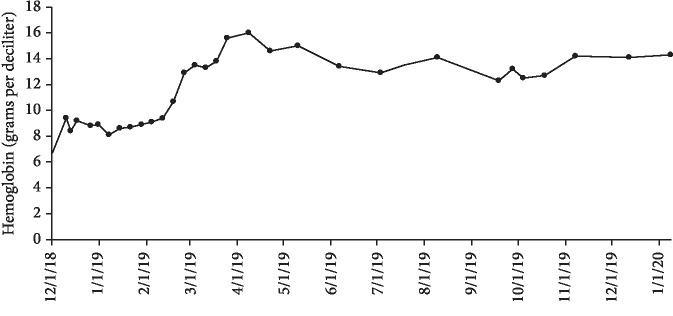
Hemoglobin recorded during Venetoclax monotherapy treatment from December 2018 to January 2020. The treatment initially began on November 28, 2018 at 100 mg and after one month was increased to 200 mg.

**Figure 3:**
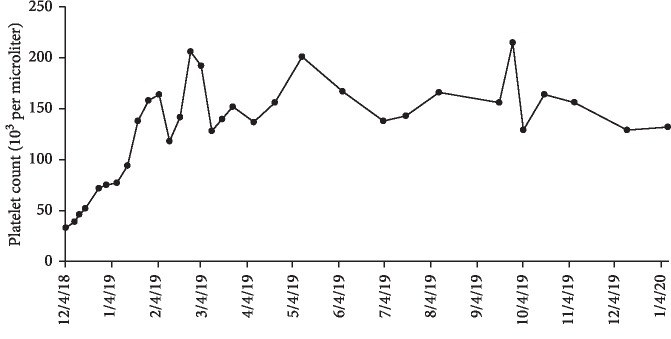
Platelet count recorded during Venetoclax monotherapy treatment from December 2018 to January 2020. The treatment initially began on November 28, 2018 at 100 mg and after one month was increased to 200 mg.

After approximately one month of 100 mg Venetoclax monotherapy, he experienced no side effects and, however, remained neutropenic and required two packed red blood cell transfusions secondary to anemia. The dose of Venetoclax was increased to 200 mg daily. The twice weekly transfusions through the VA were stopped in January 2019 and the patient is monitored via Complete Blood Count (CBC) with differential and Basic Metabolic Panel (BMP). According to his most recent appointment with Hematology on January 15, 2020, he has been transfusion-independent for twelve months. The maximum cell counts since transfusion independence was achieved as follows: white blood cell count of 3.07 × 10^9^/L, red blood cell count of 4.53, hemoglobin of 16 g/dL, and platelet count of 215,000 per microliter (Figures 1–3). His red blood cell count, hemoglobin and platelet counts are stable. No bone marrow analysis was performed after treatment with Venetoclax as the counts were improved. The patient continues to feel very well with no side effects from therapy. He remains intermittently neutropenic and, however, has not had any problems with infection.

## 3. Discussion

Hypomethylating agents are the standard treatment for myelodysplastic syndrome in the United States; however, in Europe and many countries outside the US, they are only standard of care for high-risk MDS. HMAs have been shown to prolong survival, but responses are usually transient and only 40–50% of patients respond to therapy [10]. Overall survival for patients that have lost response to HMAs is very poor at 4.3 months for high-risk MDS and 14 months for low-risk MDS [10]. MDS patients that relapse or become refractory to HMA have poor outcomes. AlloSCT is the only treatment with curative potential and, however, is associated with significant mortality, toxicity, and higher risk of relapse in patients with MDS in the context of HMA failure [11]. Currently, there are no approved standard of care options for patients with MDS that have failed initial HMAs and have contraindications to alloHSCT.

Venetoclax, a potent highly selective BCL-2 inhibitor, has been studied as a monotherapy or in combination with HMAs in MDS and AML patients. Venetoclax as a monotherapy for relapsed or refractory AML has demonstrated 19% overall response rate in heavily pretreated patients [12]. Recently, due to a phase Ib study (ClinicalTrials.gov identifier: NCT02203773), the FDA approved Venetoclax with HMAs for elderly patients (>65 years) with treatment naïve AML ineligible for chemotherapy. As a treatment for MDS, there is currently an ongoing Phase 1b, open-label, multicenter study evaluating the safety and pharmacokinetics as a monotherapy or in combination with hypomethylating agents for the treatment of relapsed/refractory myelodysplastic syndrome with an estimated completion date of August 2020 (ClinicalTrials.gov identifier: NCT02966782). A recently accepted abstract to the American Society of Hematology (ASH) 2019 Annual meeting presented preliminary results demonstrating that venetoclax in combination with HMAs led to high rates of marrow remission (55%) and hematologic improvement (38%) in a very high-risk and heavily treated MDS population after performing a retrospective review [13].

In this case, a patient with myelodysplastic syndrome refractory to current standard of care treatments with contraindications to alloSCT was treated with Venetoclax as the sole agent. After three months of treatment, the patient became transfusion independent. As of June 2019, he has not required further transfusions, however, remains mildly neutropenic. The development of new therapeutic strategies for MDS refractory to HMAs is an important avenue of research and is being extensively studied in clinical trials. Our case aims to demonstrate the feasibility of Venetoclax monotherapy for refractory MDS in hopes that in conjunction with the results of current and future clinical trials will become a standard of care option for these patients.
